# Real-Time Photometric Calibrated Monocular Direct Visual SLAM

**DOI:** 10.3390/s19163604

**Published:** 2019-08-19

**Authors:** Peixin Liu, Xianfeng Yuan, Chengjin Zhang, Yong Song, Chuanzheng Liu, Ziyan Li

**Affiliations:** School of Mechanical Electrical and Information Engineering, Shandong University, Weihai 264209, China

**Keywords:** visual SLAM, sparse direct method, photometric calibration, corner detection and filtering, loop closure detection

## Abstract

To solve the illumination sensitivity problems of mobile ground equipment, an enhanced visual SLAM algorithm based on the sparse direct method was proposed in this paper. Firstly, the vignette and response functions of the input sequences were optimized based on the photometric formation of the camera. Secondly, the Shi–Tomasi corners of the input sequence were tracked, and optimization equations were established using the pixel tracking of sparse direct visual odometry (VO). Thirdly, the Levenberg–Marquardt (L–M) method was applied to solve the joint optimization equation, and the photometric calibration parameters in the VO were updated to realize the real-time dynamic compensation of the exposure of the input sequences, which reduced the effects of the light variations on SLAM’s (simultaneous localization and mapping) accuracy and robustness. Finally, a Shi–Tomasi corner filtered strategy was designed to reduce the computational complexity of the proposed algorithm, and the loop closure detection was realized based on the oriented FAST and rotated BRIEF (ORB) features. The proposed algorithm was tested using TUM, KITTI, EuRoC, and an actual environment, and the experimental results show that the positioning and mapping performance of the proposed algorithm is promising.

## 1. Introduction

Recently, many visual simultaneous localization and mapping (SLAM) systems have been proposed, since they are fundamental building blocks for many emerging technologies, such as autonomous cars, virtual reality, and augmented reality [1]. Mobile ground equipment estimates its own position and reconstructs a three-dimensional map in real time using specific sensors without any prior environmental information [2]. 

At present, the SLAM system based on vision sensors has gained popularity in the field [3]. According to its algorithmic principle, the visual SLAM system can be divided into the direct formulation and the indirect formulation [4]. Compared with the indirect visual SLAM, the direct formulation can establish dense, semi-dense, sparse 3D reconstructions that are valuable for the navigation of ground mobile equipment [5]. In addition, research has shown that the mapping performance of the direct approach was more robust than the indirect one for the low-texture-features environment [6]. 

The direct and semi-direct formulations optimize the photometric error based on the grayscale invariant assumption to estimate the camera motion, since the sensors provide the photometric measurements [7]. J. Engel et al. [8] proposed the LSD-SLAM (large-scale direct monocular SLAM) with indirect loop closure detection based on the angular relationship between the pixel gradient and the polar line in dense reconstruction. The LSD-SLAM easily loses the tracked visual features as the camera moves quickly, since it is sensitive to the camera’s internal parameters and exposure conditions. C. Forster et al. [9] proposed SVO (semi-direct visual odometry), which is a visual odometry (VO) without back-end optimization, loop closure detection and re-localization. SVO tracks the features from accelerated segment test (FAST) feature points and surrounding pixels by minimizing the photometric error to estimate the camera motion. The speed rate of SVO can reach 100 frames per second and up to 400 frames per second in SVO2.0 [10]. To improve the robustness of the system, P. Kim et al. [11] proposed patch-based VO in 2015 using linear illumination models to compensate for the local brightness variations. Patch-based VO enhances the robustness of sudden illumination changes but has a high dependency on the scene’s textural features. J. Engel et al. [12] proposed DSO (direct sparse odometry), which is a direct pixel-tracking model with photometric parameters that calculates the residual of the pixel projection from the dominant frame to the current frame. When DSO is tracking the pixels, the system retains several key frames using a sliding window to establish the minimized energy function to obtain the pose and the inverse depth of the current camera status as the back-end [13]. In order to enhance the performance of direct visual odometry, P. Bergmann et al. [14] proposed an online photometric calibration, which dynamically estimates the photometric parameters by solving the least squares equation of the feature tracker and adjusts the exposure situation of the input sequence. It is a milestone in improving the positioning and mapping accuracy for direct formulation. Stereo DSO, which was proposed by M. Schwörer et al. [15] and improved by N. Yang et al. [16], further enhances the precise depth estimation. X. Gao et al. [17] proposed LDSO (direct sparse odometry with loop closure)—which is a SLAM system with indirect formulation loop closure detection—and evaluated it on multiple sets of datasets but not in an actual environment. In the field of direct SLAM, the computer vision group at the Technical University of Munich has made a major contribution.

**Figure 1:**
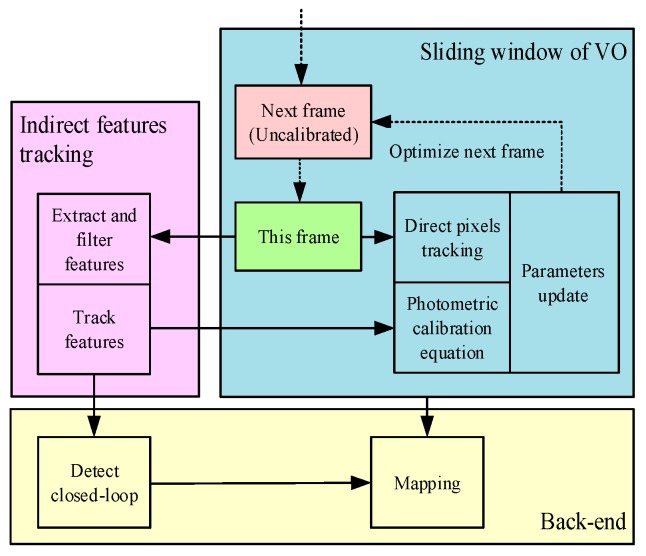
The flow chart of the proposed system. We divided the approach into three elements: The indirect feature tracker, the sliding windowed photometric compensation and the back-end optimization. The green one is the last frame that joins in the localization, mapping, and photometric parameters’ calculation. Then, the parameters were utilized to compensate the red frame based on the photometric parameters.

In the DSO series and LDSO, the photometric parameters were introduced to compensate for the vignetting and response function of input images as constants. However, the compensation, based on pre-trained photometric calibration files, could not update photometric parameters for dynamic illumination in real time. Inspired by [14] and [17], in order to further improve the robustness of the direct formulation visual SLAM system in positioning and mapping, we reinforced the LDSO algorithm by introducing real-time photometric calibration to update the exposure condition of the input sequence. In addition, a Shi–Tomasi corner filtering mechanism was designed to reduce the computational complexity of loop closure detection. The flow chart of the proposed SLAM system is shown in Figure 1. Firstly, a photometric parameter model was introduced to compensate the input sequence according to the photometric formation of the automatic exposure camera. Secondly, we utilized the robust Kanade-Lucas-Tomasi tracking method (KLT) tracker to obtain the continuous feature points between the input sequence to establish an optimization equation which integrated the KLT tracker with the direct-tracked pixels in VO. Then, the photometric calibration parameters of the visual odometry were updated in real time to optimize the exposure situation of the input sequence. Finally, a Shi–Tomasi corner filtering mechanism was introduced in the indirect back-end to realize the relocation and loop closure detection based on the ORB feature. On the generic dataset, we demonstrated that the drift error of the proposed algorithm was significantly reduced with respect to LDSO and the performance on KITTI was similar to mono-ORBSLAM. In addition, the proposed algorithm was evaluated in the actual illumination challenge environment and the experimental results indicate that the mapping performance of the proposed method is promising.

## 2. Photometric Calibration Model

According to the photometric parameters of the auto exposure camera, an optimization equation was established based on the corner point tracker. Then, the vignetting factors and the response function were dynamically updated to compensate for the input sequence illumination condition to realize the photometric calibration in real time. 

### 2.1. Vignetting and Response Function

A scene point is illuminated by a light source and reflects the energy back into space [18]. The global light intensity, which is called the radiance of the scene point, is independent of the viewing angle of the observer.

**Figure 2:**
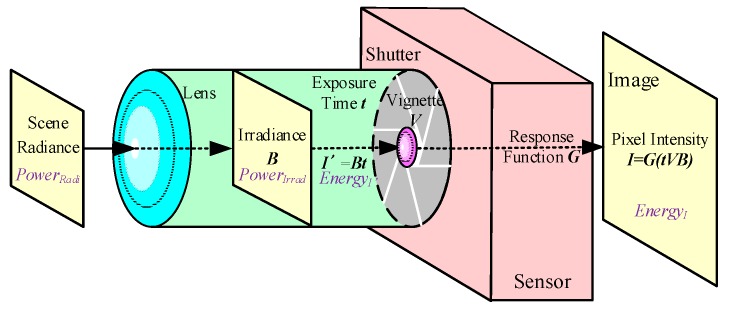
The flow chart of photometric image formation. The original energy that is emitted from the scene, which is called the radiance, is affected by the vignetting effort of the lens and the exposure time of the shutter.

When the vision sensor captures the scene as an image, the radiance of the scene’s points are converted into irradiance B by a lens. For the formation of the image each time, the total energy that is received by the sensor depends on the irradiance that passes through the camera shutter during the exposure time t. Finally, the energy turns into the pixel intensity according to the response function G [19]. The flow chart of the photometric image formation process is shown in Figure 2.

The effective incident off-axis light of the front lens was changed according to the size of the aperture and the exposure time, which caused the pixel intensity of the image to gradually weaken from the center out. In the auto-exposure mode, the adaptive exposure time was determined by the different scenes. The response function is a process through which the received photon is nonlinearly converted into a brightness value.

The imaging model of the photometric image formation in Figure 2 can be defined by Equations (1) and (2).

(1) $$I_{i}\left(x\right)=G_{i}\left(t_{i}V_{i}\left(x\right)B_{i}\left(x\right)\right)$$

(2) $$I_{i}^{\prime }\left(x\right)=t_{i}B_{i}\left(x\right)=\frac{G_{i}{}^{-1}\left(I_{i}\left(x\right)\right)}{V_{i}\left(x\right)}$$

where $I_{i}$ is the pixel intensity observed in frame i, $B_{i}$ is the power of irradiance, $I_{i}^{\prime }$ is the received energy of irradiance during once exposure time, $t_{i}$ is the exposure time, V is the lens attenuation (vignetting), and G is the response function.

Vignette V:Ω→[0,1]. Assuming that the pixel intensity attenuation factors are symmetric around the center of the image, vignetting is defined as follows [20].

(3) $$V\left(x\right)=1+\sum_{n=1}^{3}v_{n}R{\left(x\right)}^{2n}$$

where R(x) is the normalized radius of pixel x with respect to the center of the image.

Response function G:ℝ→[0, 255]. When the frames are underexposed and overexposed, their brightness values are 0 and 255, respectively. Linearization is applied to G.

(4) $$G\left(x\right)=g_{0}\left(x\right)+\overset{n}{\underset{k=1}{\sum }}c_{k}h_{k}\left(x\right)$$

The main response function $g_{0}$ and the basis function $h_{k}$ were obtained by PCA (principal component analysis). When the coefficient vector $c_{k}$ and $v_{n}$ were iteratively calculated using the photometric calibration equation, the adaptive vignetting and response function compensation of the input sequence were realized.

### 2.2. Photometric Calibration Equation

After constructing the model of the vignetting factors V and the response function G, the feature points of the images were extracted to track the input sequences to establish an optical flow equation. The equation, including the residual of the last M frames, were minimized to update the vignetting factors V and the response function G [14]. The flow of the photometric parameters’ optimization is described Figure 1.

The Shi–Tomasi corners are generally utilized as the global features to represent an image, owing to their good affine invariance. Those corners are tracked by the Kanade–Lucas (LK) optical flow on the image pyramid, which is called the KLT tracker, to construct an optical flow energy equation. 

We segmented an image into 32 × 32 regions and defined a constant of tracked candidate points to obtain a good effect. When the tracked feature was lost, a new candidate was extracted from the high-gradient region that contained fewer points. If the max gradient of a region was lower than the threshold, the region was filtered [21].

For a set of tracked points P in one frame, the proposed approach designs the function of the energy residual using its co-visual frames. The tracked pixel intensity was restored to the irradiance estimation energy during one exposure, written as ${I^{\prime }}_{i}^{p^{\prime }}$, based on the photometric formation. Then the received energy from irradiance during once exposure, written as ${I^{\prime }}_{j}^{p}$, was utilized to calculate the residual between the energy of the co-visual frames. According to Equation (2), the Huber norm of the pixel residual energy function is defined as Equations (5) and (6).

(5) $$E_{I^{\prime }}=\underset{p\in P}{\sum }\underset{i\in F_{p}}{\sum }{w_{i}}^{p}\|{I^{\prime }}_{j}^{p}-{I^{\prime }}_{i}^{p^{\prime }}{\|}_{h}$$

(6) $$r_{i}=t_{i}B_{i}-\frac{G_{i}{}^{-1}\left(I_{i}^{p}\right)}{V_{i}^{p}}$$

$F_{p}$ is the set of frames that can observe the points P. The photometric parameters were dynamically chosen by minimizing Equation (5) using the L–M approach. The optimization process of the photometric parameters is described in detail in Section 3.

**Figure 3:**
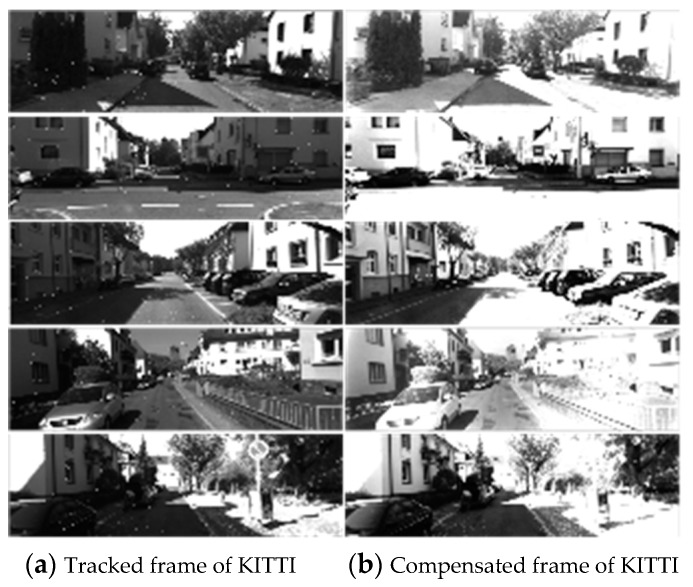
The partial photometric calibration results of KITTI sequence 00. The subfigures (**a**) are the tracked original frames, and the subfigures (**b**) are the compensated frames. It can be seen in subfigure (**b**) that the global exposure was enhanced, especially at the edge of the image. In addition, the brightness values of subfigure (**b**) remained continuous.

The estimation of the vignetting and response functions requires multiple images, which are difficult to collect in time during one calibration. Therefore, the states of the current vignetting and response function were maintained after the current estimation to compensate the last frame. Then, the compensated frame was used for localization, mapping, and evaluating the photometric parameters in the next frame, as shown in Figure 1. The adaptive photometric compensation results of the dataset were randomly selected, as shown in Figure 3.

## 3. The Combination of Photometric Calibration and Direct SLAM

In the direct formulation, the minimized photometric error was utilized to achieve the camera pose based on the grayscale invariance assumption. DSO integrates the photometric parameters to simulate the vignetting effect and the gamma attenuation to enhance the robustness of VO. However, the scene radiance of VO was calculated using the pretrained photometric calibration files that were proposed in [1].

To further enhance the performance of the direct formulation, the photometric calibration should adapt to a continuous pixels’ brightness value to respond to the illumination challenge. When the last frame enters the sliding window, it is compensated based on the previous frames and then applied to the front-end of the SLAM system.

### 3.1. Direct Sparse Model

After the frame is compensated, it enters the VO model to join the localization and mapping. The covisual pixels in all frames of the sliding window are projected onto the current frame to build the photometric error equation, which is given by Equation (7).

(7) $$minE_{photo}=min\underset{i\in F_{w}}{\sum }\underset{p\in P_{i}}{\sum }\underset{j\in obj(p)}{\sum }E_{p_{j}}$$

where $F_{w}$ is the set of all frames in the sliding window, $P_{i}$ is the set of all observed pixels on the host frame, and obj(p) is the set of co-visual frames that can observe the pixel p. The estimation of the inverse depth and camera pose were achieved by minimizing Equation (7), which can be shown as the flowing factor graph [12].

There are at most $N_{f}$ active key frames in each sliding window. When a new frame enters the sliding window, it is tracked to determine whether to create a new key frame. After obtaining enough key frames, the redundant key frames are deleted according to the marginalized strategy to reduce the calculation costs [7,12].

In Section 2.2 of Chapter 2, the input image was divided into 32 × 32 regions. The pixel tracking only happens where the maximum pixel gradient is greater than the threshold. If the value of the threshold of each region equals the average gradient, then one a constant must be added.

### 3.2. Parameters Update

The tracking of the indirect feature, which was utilized to update the response function and the vignetting factor, was simultaneously performed with the estimations of the inverse depth and the camera pose in the sliding window. Thus, the tracking equations in the sliding window are rewritten as Equations (8) and (9).

(8) $$r_{i}=t_{j}B_{j}-e^{-a_{i}}\frac{G^{-1}\left(I_{i}^{p^{\prime }}-b_{i}\right)}{V_{i}}$$

(9) $$E_{p_{j}}=\underset{p\in N_{p_{k}}}{\sum }w_{p}\|r_{i}{\|}_{h}$$

where $N_{p_{k}}$ epresents the neighboring pixels of pixel $p_{k}$; $t_{i}$ and $t_{j}$ are the exposure times of images $I_{i}$ and $I_{j}$, respectively; a and b are the affine brightness transform parameters [22]; and $p^{\prime }$ is a reprojection pixel of p on $I_{j}$. Combined with Equations (3) and (4), we set x as the total number of variable parameters to optimize Equation (9).

(10) $$x={\left[\xi ,a,b,c,v\right]}^{T}$$

$\xi \in {\mathbb{R}}^{6}$ the camera state, c=($c_{1},c_{2},c_{3},c_{4}$) is the coefficient vector of the response function G, and v=($v_{1},v_{2},v_{3}$) is the vignetting coefficient vector. Considering that the exposure time t can be estimated by two consecutive frames, the proposed approach suggests decoupling the exposure time t estimation from the other parameters [14]. According to Equations (8) and (9), the visual odometry with the adaptive exposure compensation equation is introduced as Equation (11).

(11) $$minE_{CalibVo}=min\underset{i\in F_{w}}{\sum }\underset{p\in P_{i}}{\sum }\underset{j\in obj(p)}{\sum }E_{p_{j}}$$

The L–M algorithm is applied to calculate the Jacobian matrix of the residual $r_{i}$ as

(12) $$J_{x_{i}}=\frac{\partial r_{i}}{\delta x_{i}}=\left(\frac{\partial r_{i}}{\delta \xi_{i}},\frac{\partial r_{i}}{\delta a_{i}},\frac{\partial r_{i}}{\delta b_{i}},\frac{\partial r_{i}}{\delta c_{i}},\frac{\partial r_{i}}{\delta v_{i}}\right)$$

(13) $$H\delta x_{i}=-b$$

(14) $$H={J_{x_{i}}}^{T}W_{x_{i}}J_{x_{i}}+\lambda I$$

(15) $$b={J_{x_{i}}}^{T}W_{x_{i}}r_{i}$$

Equations (12)–(14) describe the iterative solution process of Equation (11) based on the L–M approach. Here, the weight matrix $W_{x_{i}}$. was inversely proportional to the image gradient of $x_{i}$ [12]. 

**Figure 4:**
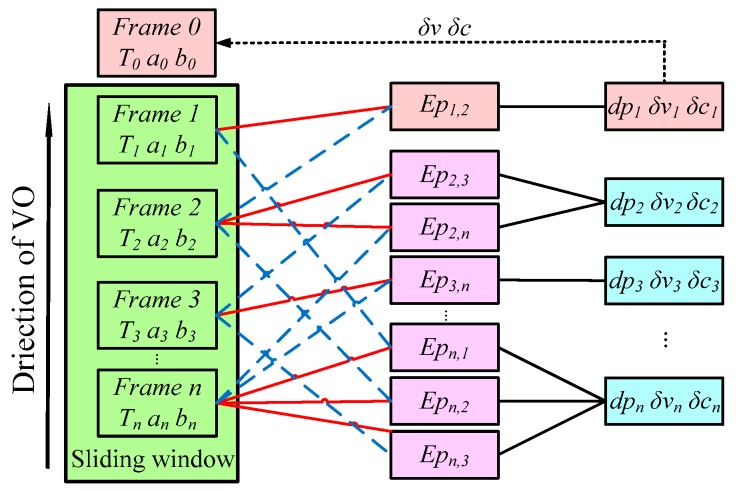
The factor graph of the direct formulation. The tracked pixel of the host frame is represented by the solid red line, which is linked to co-visual frames by the dotted blue line. For each term of the tracked pixel, an energy function of the residual was established to calculate the inverse depth and photometric parameters, which are shown by the black line. Then, the parameters were utilized to compensate the next frame.

By solving δx=(δξ,δa,δb,δc,δv), the vignetting V and the response function G were updated to realize the real-time photometric calibration of the input sequence. Figure 4 shows that when a new frame $I_{i}$ arrived, its response function and vignetting, which were calculated based on the last $F_{w}$ frames, were removed to restore the scene’s radiance. The system maintains the previous vignetting and response function state to restore the current frame $I_{j}$, and simultaneously updates the previous vignetting state and response function. Then, the incoming frames are calibrated based on the last response function and the vignetting estimation.

### 3.3. Window Optimization

**Figure 5:**
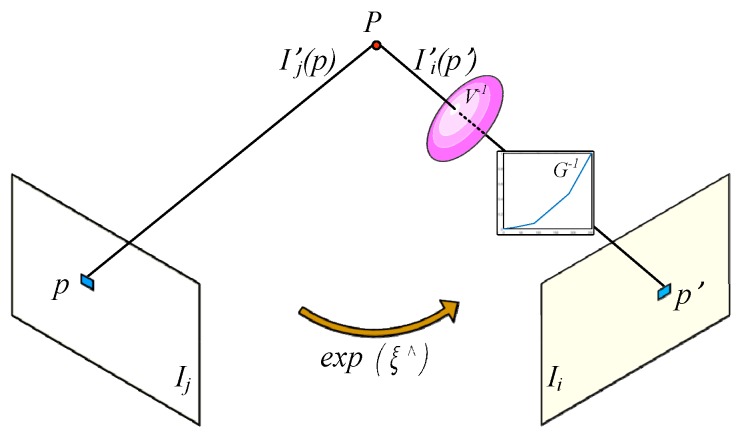
The photometric error based on the photometric formation. The pixel intensity of tracked point p, which is called $p^{\prime }$, was restored to the estimated scene radiance and then the residual with the current scene radiance was calculated to establish the photometric error equation. For the camera pose change between $I_{j}$ and $I_{i}$, the pose equation based on the locations of $p^{\prime }$ and p was utilized to calculate the se(3).

When a new key frame is inserted, the current sliding window is optimized using the bundle adjustment (BA) [7] like the local loop closure of ORBSLAM [23,24] to reduce the drift localization error, as shown in Figure 5.

In Figure 5, $\xi \in {\mathbb{R}}^{6}$ includes the element of se(3), and ξ^ is the anti-symmetric matrix of ξ. For all keyframes $F_{wkey}$ in the sliding window, the camera pose optimization equation is established as follows.

(16) minEwkey=min∑i∈Fwkey∑p∈Pi‖p′ −1siKexp(ξ^)p‖22

The least squares problem represented by Equation (16) can be iteratively solved using the L–M algorithm, and then the best current camera pose ξ^ can be obtained. The graph optimization was based on g2o library [25].

### 3.4. Loop Closure Detection

**Figure 6:**
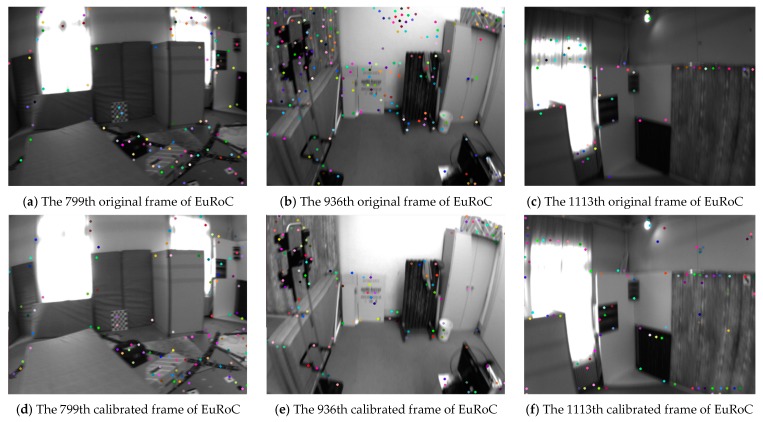
The experimental results on the EuRoC V1_03_difficult dataset. Subfigures (**d**), (**e**) and (**f**) are modified from subfigures (**a**), (**b**) and (**c**) respectively.

In LDSO, the loop closure detection was realized by calculating the ORB descriptor of the tracked Shi–Tomasi corner. However, the tracked candidate points may be extracted in the low gradient region, which increases the calculation burden and affects the performance of the loop closure detection [26]. In the proposed approach, a corner extraction and screening strategy was designed so that the 32 × 32 regions were segmented in each image and the low-gradient regions were screened, as in Section 2.2 of Chapter 2. When the tracked features are lost, new candidate points will be extracted from the smaller region and the total number of points is constant. This strategy improved the localization performance of the system while enhancing the effective calculation capability. The results of the robust Shi–Tomasi corner detection on the EuRoC [27] V1_03_difficult dataset are shown in Figure 6.

As can be seen from Figure 6, the number, the distribution and the area texture of the detected features are obviously adjusted. With the help of photometric calibration and the Shi–Tomasi corner filtering mechanism, the features in the low texture regions (e.g., the wall in Figure 6b,e) are filtered and the features in the high texture regions (e.g., the cabinet in Figure 6c,f) are increased. In the loop closure detection process, DBoW3 was used to build the database of the bag of words (BoW) model to achieve loop closure detection [28]. The corresponding descriptors are re-coded based on the pixels’ intensity around the features. It can be concluded that the stable quantity and reasonable distribution of the tracked features and their descriptors can make the score—which is calculated by the BoW model— become more reliable. Then the tree node of the marginalized key frames in the BoW model are selected to realize the loop closure detection. Thereafter, the loop closure detection performance of the proposed system can be facilitated. The loop closure detection experiment of the proposed system and the LDSO can be seen in Section 4.1.

## 4. Experiments

The proposed algorithm was operated on a laptop with an Intel i7-8750H CPU, 16G of memory, and Ubuntu16.04. GPU acceleration was not adopted during the experiments. The simulation and the actual experiments were designed to evaluate the localization accuracy, loop closure performance, and point cloud map of the proposed algorithm.

The experimental designs were grouped according to the dataset in this paper. Firstly, the localization accuracy, the photometric parameters ‘calibration and the pixels’ tracking performance were tested on the TUM dataset [29]. Secondly, the localization accuracy, the timing cost and the loop closure detection performance were evaluated on the KITTI [30] dataset. Thirdly, the feature detection of loop closure and point cloud map were shown on the EuRoC challenging illumination dataset. Finally, the proposed method was tested in a custom environment where the illumination in the room was being changed under control.

### 4.1. Experiments Based on Different Datasets

**Figure 7:**
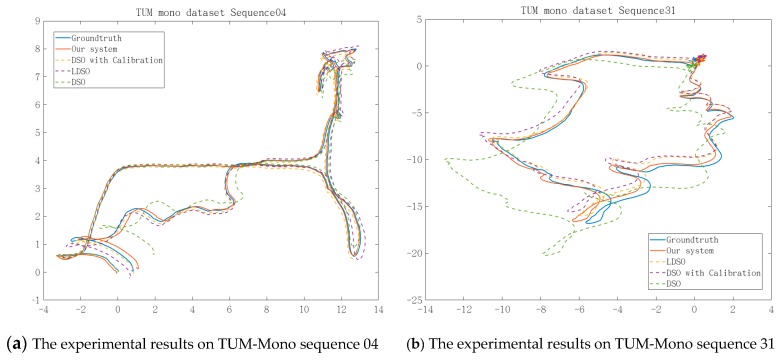
The experimental results on the TUM-Mono dataset. Subfigures (**a**) and (**b**) show the trajectories of sequences 04 and 31, respectively, along the *x*-axis and *z*-axis on our system. Down are direct sparse odometry (DSO); DSO with loop closure (LDSO); and enhanced DSO, which was integrated with the algorithms proposed in [12] and [14].

The proposed algorithm was evaluated using the TUM-Mono and KITTI Odometry datasets in a monocular setting. The TUM dataset [29] is a scenic dataset of the Technical University of Munich, including 50 laboratory and outdoor sequences. The proposed algorithm was tested using the TUM-Mono dataset sequences 04 and 31, and compared the localization accuracy with the original DSO [12], the original LDSO [17], and the enhanced DSO facilitated by the online photometric calibration [14], respectively. The experimental results are illustrated in Figure 7.

Figure 7 shows that the trajectories obtained by the online photometric calibration-enhanced DSO [14] are evidently better than that of the original DSO [12]. However, the performance was limited due to the lack of loop closure detection. As Figure 7 shows, our approach corrected the partially distorted segments of the LDSO and obtained the best overall performance among DSO, LDSO, and the enhanced DSO [14], due to the loop closure detection which was improved by the adaptively compensated pixel intensity. 

**Figure 8:**
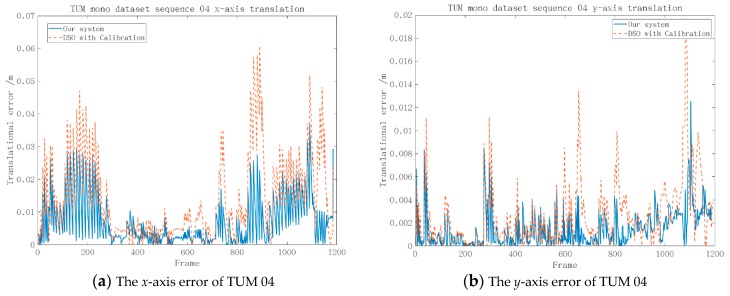

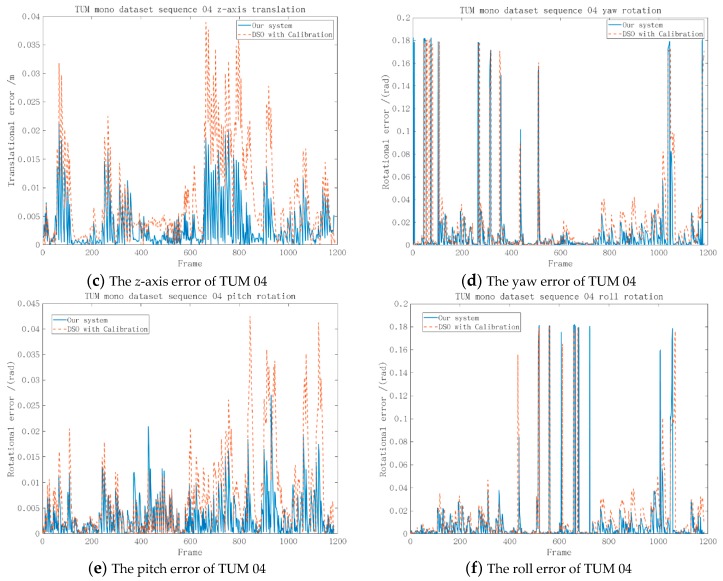
The error of the 6-degree of freedom (6-DoF) on TUM-Mono sequence 04 with respect to the ground truth between our system and the enhanced DSO [12] and [14].

As shown in Figure 8, we calculated the errors of the translation and the Euler angle transformation with respect to ground truth. In the subfigures, the residuals were controlled within a reasonable range.

Figure 8a–c demonstrates that the proposed system had smaller errors along the *x*-axis, *y*-axis, and *z*-axis compared with the enhanced DSO [14] and the errors of our method stayed within a reasonable range on TUM sequence 04. However, Figure 8 d–f indicates that the proposed algorithm had a similar performance in the Euler angle transformation of the enhanced DSO [14].

**Figure 9:**
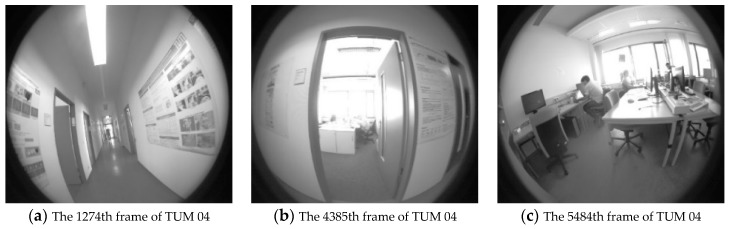

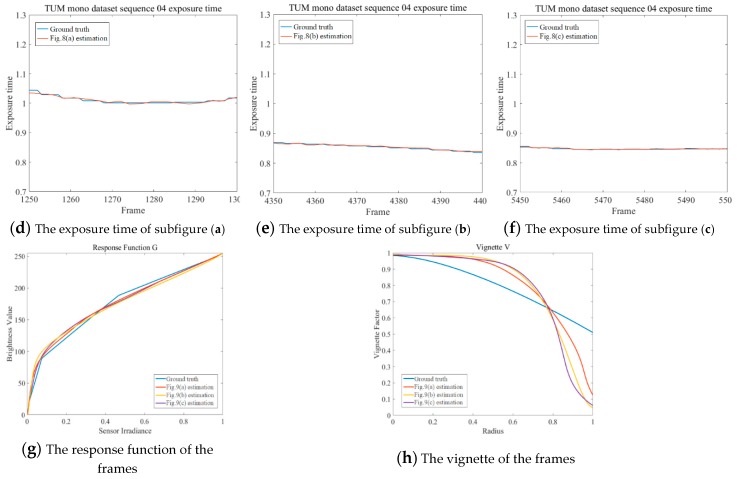
The photometric parameters of randomly selected frames. It can be seen that the estimated exposure times were very close to the ground truth. However, the parameters of response function and vignette were acutely adjusted to fit the pixels’ intensity of the different scenes.

The photometric parameters of randomly selected frames were calculated and are shown in Figure 9. The vignette and response function were dynamically estimated when the irradiance function had accumulated to a reasonable range while the exposure time could be estimated frame by frame. The results show that the estimated exposure times were closer and closer to the ground truth as the frame number increased, and the response function and vignette were dynamically adjusted around the ground truth to fit the different exposure conditions.

As can be seen in Figure 9, the vignette and response function were dynamically estimated when the irradiance function had accumulated to a reasonable range, while the exposure time was estimated frame by frame. The results show that the estimated exposure times were closer and closer to the ground truth in the frames across time, and the response function and vignette were dynamically adjusted around the ground truth to fit the different exposure conditions. 

**Figure 10:**
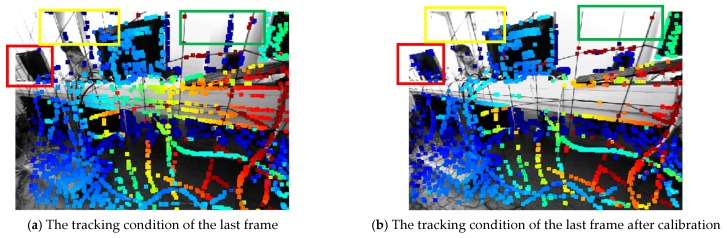
The exposure condition and pixel tracking of TUM mono dataset sequence 04.

The last frame of TUM mono dataset sequence 04 was captured to compare the condition of exposure and tracking performance after photometric calibration and the experimental results are shown in Figure 10.

Figure 10a is the original frame and Figure 10b is the frame after photometric calibration. As can be seen, there are two main differences. Firstly, the exposure of Figure 10b was enhanced. The global brightness and pixel contrast were obviously improved by the adaptive response function and vignette. Secondly, the tracked pixels in the low texture region were filtered like the window at the upper part of the image (regions in yellow and green boxes) and the pixels in the high texture region were increased, like the computer at the middle part of the image (region in red box). The enhancement was set to improve the robustness of the tracking, which would then promote the depth estimation accuracy of visual odometry.

To further verify the performance, the KITTI dataset was utilized to test the localization accuracy, the loop closure detection performance, and the timing cost of the proposed algorithm. The KITTI dataset [30] was jointly produced by the Karlsruhe Institute of Technology in Germany and the Toyota Institute of Technology in the United States. It is currently the largest computer vision algorithm evaluation dataset in the world for autonomous driving scenarios. 

**Figure 11:**
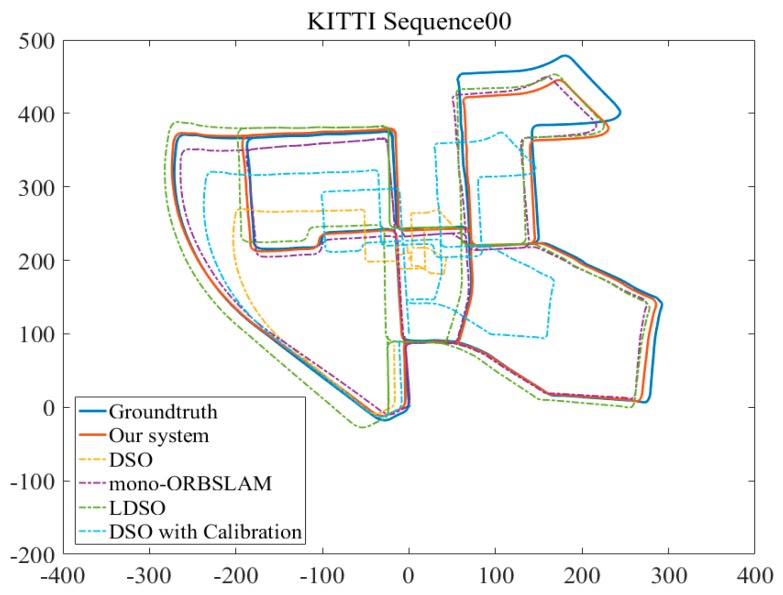
The experimental trajectories results of our system, DSO, LDSO and the enhanced DSO which was integrated with the algorithms proposed in [12] and [14] along the *x*-axis and *z*-axis of the KITTI dataset sequences 00.

In the evaluation of [15], monocular VO was considered to be unusable for such a large-scaled dataset, which was overcome by introducing loop closure detection in [17]. Therefore, the 00-10 sequences of the KITTI dataset were applied to test the proposed algorithm. As shown in Figure 11, the experimental results were compared with DSO [12], LDSO, the enhanced DSO [14] and mono-ORBSLAM [24]. 

The trajectories along the *x*-axis and *z*-axis were recorded in Figure 10. The experimental results of the proposed method for KITTI sequences 00-10 were better than those of DSO, LDSO and had a similar performance to mono-ORBSLAM. In addition, we also evaluated the performance of the enhanced DSO [14] on KITTI sequence 00. Evidently, the trajectory of the enhanced DSO was distinctly corrected with respect to the DSO’s result, and it could almost close the loop. However, the correction was limited due to the lack of an excellent loop closure detection strategy.

At present, the SLAM algorithm’s performance indicators mainly include the ATE (absolute trajectory error) and RPE (relative position error). The ATE was utilized to compare the localization accuracy of the SLAM algorithm due to its directness. We calculated the ATEs of DSO, LDSO, and the proposed algorithm on KITTI and collected them in Table 1.

**Figure 12:**
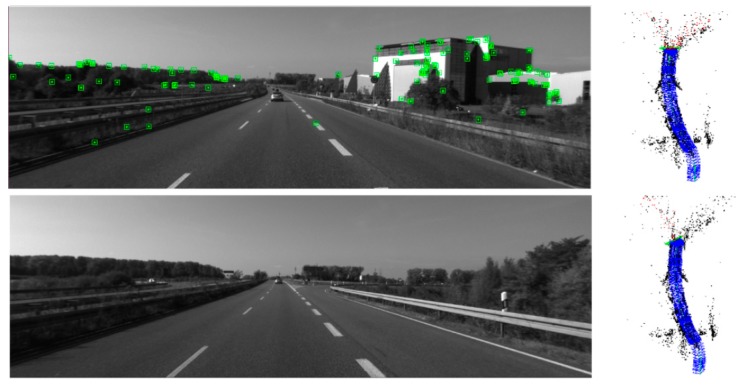
The lost ORB features during tracking on the sequence 01 of KITTI dataset.

The ATE of mono-ORBSLAM could not be obtained because of the tracking failure around the 585th frame of the KITTI dataset sequence 01 as Figure 12. 

The experimental results in Table 1 show that the proposed algorithm had a better positioning performance on most KTITI sequences and had similar performance to that of mono-ORBSLAM [24]. However, the loop closure detection performance of LDSO in sequence 09 declined because of the repeated frames around the loop closure that were too small to detect. When the previous bright frames participated in compensating the frames around the loop closure based on the photometric formation, the repeated images became much brighter than before. However, those frames were dark at the initial stage of the sequence. Therefore, after the compensation, the global brightness difference around the loop closure reduced the positioning performance. 

**Figure 13:**
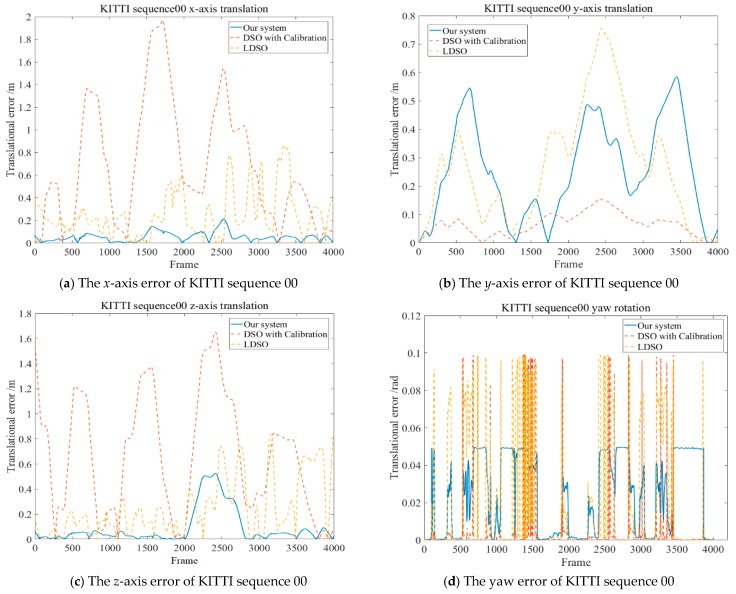

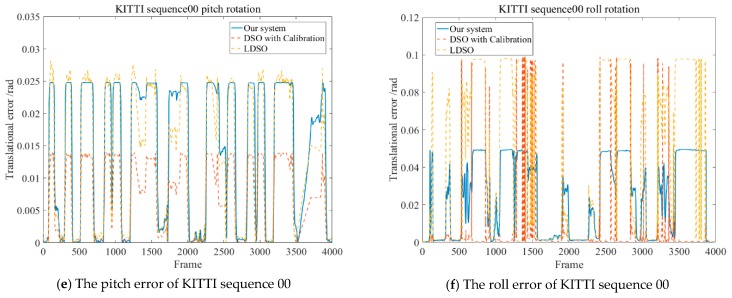
The residuals of the 6-DoF on KITTI sequence 00 including the translations and Euler angle of rotations. The residuals of subfigure (**b**) were obviously larger in the both proposed system and LDSO because of the introduction of loop closure detection. However, the error tendencies on *x*-axis and *z*-axis were primarily lower than LDSO and the enhanced DSO [14]. In general, the translation error and rotational error of the proposed system were stably maintained as reasonable values along all frames of KITTI sequence 00.

The 6-DoF rigid body motion error was calculated, as shown in Figure 13, to demonstrate the performance along the frames related to LDSO. The unit of the translational error is meters, and the unit of the rotational error is radians.

Figure 13 shows that the error of the proposed system with respect to ground truth was in a reasonable range and better than the enhanced DSO [14]. The max translational residuals along the *x*-axis and the *z*-axis were 0.2153 m and 0.5259 m, respectively, and the rotational error of the camera pose was less than 0.05 rad, which further proves that the proposed system can better cope with the illumination changes in the KITTI dataset sequence 00.

**Figure 14:**
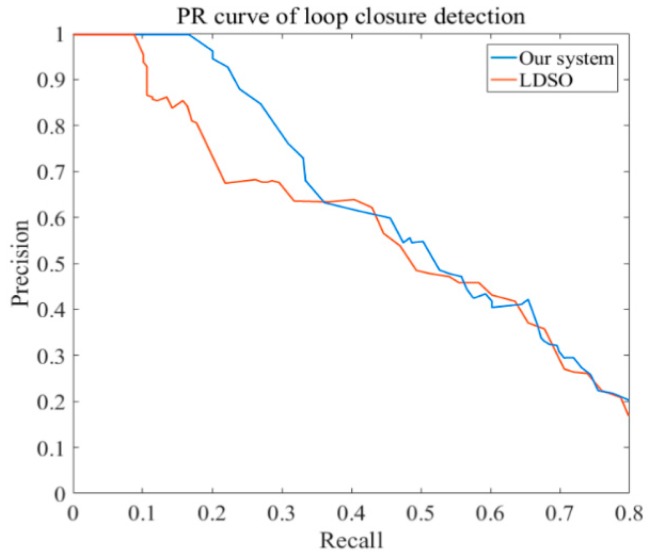
The contrast of precision-recall ratios between our system and LDSO on KITTI dataset.

The precision–recall (PR) curve is widely applied to evaluate the performance of loop closure detection. The percentage of loop closures, which were correctly detected in all the detections, was represented as the precision ratio. The percentage of loop closures, which were correctly detected in all real loop closures, was defined as the recall ratio. To compare the loop closure detection performance of the proposed system and LDSO, the PR curve of the loop closure detection is illustrated in Figure 14, from which we can see that when the recall ratio equals 0.5, the precision ratio of our system is larger than that of LDSO. In addition, the proposed system has a higher max recall ratio when the precision ratio equals 1. Figure 14 indicates that the proposed system has better loop closure detection performance compared with LDSO.

The research in this paper was mainly based on the improvement of LDSO [17] and online photometric calibration [14]. With the introduction of real-time photometric calibration, the average processing costs of a single-frame image for KITTI sequences 00-10 are provided in Table 2.

The experimental results show that the real-time photometric calibration direct SLAM system can obtain a 19.7% higher accuracy performance and 4.7% bigger timing costs than LDSO. Due to the performance improvement of the direct SLAM, the extra time burden is acceptable.

**Figure 15:**
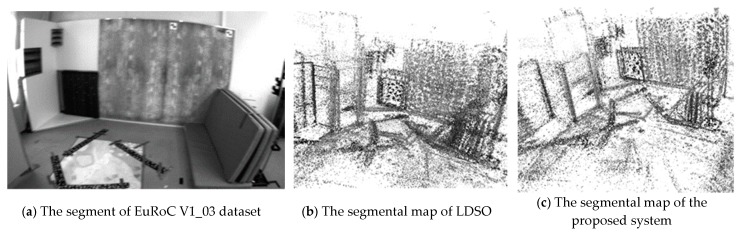
The segmental experimental results of LDSO and proposed system on the EuRoC dataset, V1_03_difficult sequence.

The EuRoC micro aerial vehicle datasets [27] were produced by the Swiss Federal Institute of Technology Zurich, including stereo images and inertial measurement unit (IMU) data. In order to analyze the limits of the proposed system, the point cloud maps of our system and LDSO on the EuRoC dataset, V1_03_difficult challenging illumination sequence, are segmentally shown in Figure 15.

As can be seen from Figure 15c, the point cloud map has less noise than Figure 15b. The online photometric calibration can improve the performance of mapping by compensating the exposure condition; however, the effect is not very satisfying. The exposure condition of the image becomes unstable due to the overdue response function and vignetting. The instability may be attributed to the KLT tracker’s intrinsic sensibility to illumination change and the blurry images, which were captured in the fast-changing scenes by a violent shaking micro aerial vehicle. To further improve the robustness of tracking, the indirect feature matching, the more robust descriptors, and the deblurring strategy, can be tested in online photometric calibration for future work.

### 4.2. Actual Experiment

We emphatically evaluated the proposed algorithm in an actual environment to prove the enhancement related to LDSO. To collect the real-time images, the Basler acA1920-155uc camera was selected, which is a global shutter complementary metal oxide semiconductor (CMOS) industrial camera produced by Basler, Germany. It has a 1920 × 1200 maximum resolution and a 164 fps maximum rate. 

**Figure 16:**
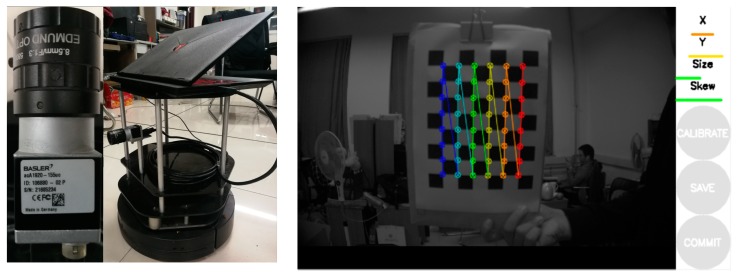
The camera and notebook were installed on the mobile ground equipment. Then, the camera was calibrated using a checkerboard to eliminate radial distortion.

The camera and notebook are equipped on a TurtleBot2, which is a robot operation system (ROS)-based mobile research platform that was produced by YUJIN, Korea. The experimental platform and the camera calibration are shown in Figure 16.

To achieve better real-time performance, the adopted resolution was 640 × 480 during the experiment, which can be modified in the calibration file of the camera. 

**Figure 17:**
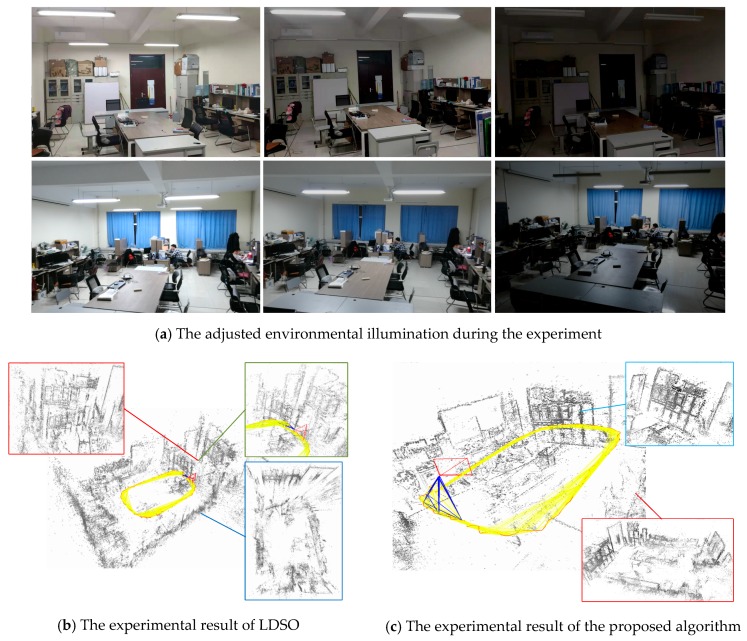
The actual environmental experiment of LDSO and the proposed algorithm. Subfigure (**a**) shows the adjusted environmental illumination during the experiment. Then, we tested the LDSO and proposed algorithm with respect to subfigure (**a**).

After the preparation of the experiment, the ROS (Robot Operation System, ROS) was utilized to control the ground equipment to acquire the scene’s visual information and perform positioning and mapping. The process of the experiment was recorded as Figure 17.

In Figure 17a, after the initialization of the camera, the light source of the experimental scene was successively adjusted. We gradually reduced the brightness of the laboratory during the equipment by moving around. The localization and mapping effects of LDSO and the proposed algorithm are shown in subfigures Figure 17b,c, respectively. In Figure 17b, the point cloud of the cabinet was repeated as the green part, and the scene splicing was distorted as the blue part. In Figure 17c, the distorted construction of Figure 17b was calibrated. The comparison between Figure 17b and 17c shows that the construction of the point cloud map was greatly affected by the exposure of the scene. When the adaptive exposure compensation parameters are not introduced in the direct visual SLAM, the brightness of the input sequence is discontinuous, which causes deviations in localization and mapping. Therefore, the proposed visual SLAM overcame the discontinuous brightness of the scene using adaptive compensation to calculate a more robust point cloud map and camera pose estimation.

## 5. Conclusions

In this paper, a real-time photometric calibrated monocular direct visual SLAM was proposed to dynamically compensate for the input sequence’s exposure. It solved the problem that the LDSO had—poor positioning and mapping robustness, due to illumination challenges. The enhanced sparse direct visual SLAM formulation was more suitable for the research and application of navigation and positioning in mobile ground equipment. Firstly, the vignetting and response function according to the photometric formation were introduced into the front-end. Secondly, the Shi–Tomasi corners of the input sequence were filtered and added to the tracking optimization equation using the tracked pixel in visual odometry. Then, the L–M approach was utilized to iteratively calculate the photometric parameters in the sliding window to compensate for the exposure condition of the input sequence. Finally, the tracked Shi–Tomasi corners in the adaptive photometric calibration and their ORB features were applied to achieve loop closure detection. The results of multiple simulations and experiments show that the proposed method had a better positioning and mapping performance than DSO and LDSO. In particular, the DSO which was integrated with the online photometric calibration, was also complementally tested to prove the generalization performance of the algorithm [14] to a certain extent, and further illustrated the advantages of our algorithm. The positioning accuracy and the point cloud map’s clearness from the proposed system, in most sequences of the KITTI and TUM-Mono datasets, were better; and the performance of our system on KITTI was similar to mono-ORBSLAM. In the actual experiment, the proposed approach was evaluated using an artificial dynamic illumination environment. As in the simulation experiment, we still obtained better positioning and mapping effects on both the TUM and the KITTI datasets than LDSO.

In future work, we will consider enhancing the adaptability of the online photometric calibration to further calibrate the visual odometry. The insertion mechanism of the key frames and the loop closure strategy will be adjusted to improve the calculation efficiency. In addition, we will seek to introduce semantic information into the direct SLAM to achieve a better loop closure detection.

## Figures and Tables

**Table 1:** The absolute trajectory errors (ATE) of our system, DSO, LDSO and ORB-SLAM on KITTI.

Sequence	DSO [17]	LDSO [17]	ORBSLAM [17]	Our System
00	126.7	9.322	8.27	**7.48**
01	165.03	**11.68**	-	20.15
02	138.7	31.98	26.86	**12.14**
03	4.77	2.85	**1.21**	2.04
04	1.08	1.22	0.77	**0.13**
05	49.85	5.1	7.91	**5.09**
06	113.57	13.552	12.54	**11.08**
07	27.99	2.96	3.44	**0.56**
08	120.17	129.02	**46.81**	105.4
09	74.29	**21.64**	76.54	26.90
10	16.32	17.36	**6.61**	17.45

**Table 2:** Timing results of our system and LDSO.

System	Sections	Time
LDSO	Total	894.43ms
Our system	Filtering and tracking feature	40.21 ms
	Exposure time estimation	3.24 ms
	Parameters ***v*** and ***c*** update	193.15 ms
	Input frame *I* optimization	135.23 ms
	Back-end	564.51 ms
	Total	936.34 ms
